# Seroepidemiology of *Toxoplasma gondii* Infection in Psychiatric Inpatients and Healthy Controls in North Iran: A Case–Control Study

**DOI:** 10.1155/japr/2530719

**Published:** 2026-10-14

**Authors:** Hamidreza Zakerzadeh Khoshroudi, Najmeh Shahini, Rasool Mohammadi, Farideh Tohidi, Aieneh Hagieh Pangh, Seyed Mohammad Mousavi, Mitra Sharbatkhori

**Affiliations:** ^1^ Student Research Committee Golestan University of Medical Sciences Gorgan Golestan Iran goums.ac.ir; ^2^ Birmingham and Solihull Mental Health NHS Foundation Trust Birmingham UK; ^3^ Clinical Research Development Unit (CRDU) Agh ghala Hospital Golestan University of Medical Sciences Gorgan Iran goums.ac.ir; ^4^ Nutritional Health Research Center Health and Nutritional Department Lorestan University of Medical Sciences Khorramabad Iran lums.ac.ir; ^5^ Laboratory Sciences Research Center Golestan University of Medical Sciences Gorgan Golestan Iran goums.ac.ir; ^6^ Parasitology and Mycology Lab School of Medicine Golestan University of Medical Sciences Gorgan Golestan Iran goums.ac.ir; ^7^ Research Center for Hydatid Disease Kerman University of Medical Sciences Kerman Kerman Iran kmu.ac.ir; ^8^ Infectious Diseases Research Center Jorjani Clinical Sciences Research Institute Golestan University of Medical Sciences Gorgan Golestan Iran goums.ac.ir

**Keywords:** bipolar disorder, case–control study, psychiatric disorders, schizophrenia, seroepidemiology, *Toxoplasma gondii*, toxoplasmosis

## Abstract

**Objective:**

Gorgan, in northern Iran, is a humid region where *Toxoplasma gondii* is common. The association with psychiatric illness has never been examined there. We measured seroprevalence in psychiatric inpatients and matched controls, quantified it for each major DSM‐5 diagnostic category, and identified local risk factors for infection.

**Method:**

A case–control study was conducted with 100 psychiatric inpatients and 100 healthy controls, frequency‐matched 1:1 on sex and 5‐year age stratum. Diagnoses were recorded as free text at discharge by the attending psychiatrists under DSM‐5 criteria and were mapped to six categories using rules fixed in advance. Sera were tested for anti‐*T*. *gondii* IgG and IgM by ELISA. Associations were estimated by logistic regression, with secondary analyses comparing each diagnostic category with the controls.

**Results:**

Anti‐*T*. *gondii* IgG seroprevalence was 39.0% in patients and 47.0% in controls (OR = 0.721, 95% CI: 0.411–1.265, *p* = 0.254; adjusted for the matching variables, OR = 0.668, 95% CI: 0.359–1.240, *p* = 0.201); IgM positivity was rare (1.0% vs. 0%). No diagnostic category differed from controls. Seroprevalence was 38.9% in schizophrenia spectrum and other psychotic disorders (*n* = 18; OR = 0.73), 34.8% in bipolar and related disorders (*n* = 23; OR = 0.62), 36.4% in depressive disorders (*n* = 22; OR = 0.66), and 57.9% in substance‐ or medication‐induced disorders (*n* = 19; OR = 1.52). Heterogeneity across categories was not significant (*p* = 0.533). Psychiatric status did not predict seropositivity (aOR = 0.85, 95% CI: 0.42–1.70). Age was the only consistent independent predictor (aOR = 1.08 per year, 95% CI: 1.04–1.11, *p* < 0.001), seroprevalence rising from 5.0% at 20 years or younger to 72.2% above age 50.

**Conclusion:**

Latent toxoplasmosis was not associated with psychiatric disorder overall or within any diagnostic category. Seroprevalence was governed principally by cumulative age‐related exposure. These data add to a growing set of null and inverse reports from high‐endemicity settings and indicate that the epidemiological expression of this association is context‐dependent.

## 1. Introduction

*Toxoplasma gondii* is an obligate intracellular protozoan parasite and one of the most successful pathogens worldwide, with an estimated one‐third of the human population carrying a latent infection [1, 2]. Felids, particularly domestic cats, are the definitive hosts in which sexual reproduction occurs, and they shed environmentally resistant oocysts in their feces [3]. Once sporulated, these oocysts are infectious to humans and other warm‐blooded intermediate hosts [4].

Humans acquire infection by three routes: ingestion of tissue cysts containing bradyzoites in raw or undercooked meat, vertical transmission from a newly infected mother to her fetus, and ingestion of food, water, or soil contaminated with sporulated oocysts. The environmental route matters most epidemiologically, since oocysts remain viable in moist soil and water for months to years and form a persistent reservoir [5–7]. Seroprevalence therefore varies with climate, diet, and sanitation [8, 9], and hot, humid regions that favor oocyst survival tend to report higher rates. Iran is a high‐prevalence country, with national seroprevalence estimated at approximately 40% [4, 10, 11]. Local environmental conditions are central to how the parasite is transmitted there.

After an acute infection that is usually asymptomatic in immunocompetent individuals, *T*. *gondii* enters a lifelong chronic phase in which tissue cysts form, mainly in the central nervous system and muscle [12, 13]. The parasite shows strong tropism for the brain, where it persists in neurons and glial cells [13]. These cysts were long considered dormant. They are in fact metabolically active and can modulate host neurobiology through several interconnected mechanisms [14].

The first mechanism is direct dysregulation of dopaminergic pathways: The parasite genome encodes a tyrosine hydroxylase homolog, the rate‐limiting enzyme of dopamine synthesis, which may contribute to the hyperdopaminergic states associated with psychosis [12, 13]. The second is sustained, low‐grade neuroinflammation. Persistence in the central nervous system triggers microglial activation and local production of proinflammatory cytokines, including interferon‐gamma (IFN‐*γ*), interleukin‐6 (IL‐6), and tumor necrosis factor‐alpha (TNF‐*α*). This matters because the inflammation hypothesis of mental illness holds that chronic neuroinflammation contributes to neuronal damage and neurotransmitter dysregulation in both schizophrenia and mood disorders [15–18]. The third mechanism connects the immune response to neurotransmitter metabolism through the kynurenine pathway. IFN‐*γ* produced in response to the parasite activates indoleamine‐2,3‐dioxygenase (IDO), which catabolizes tryptophan and so depletes the precursor of serotonin, while shunting tryptophan metabolism towards neuroactive kynurenine metabolites that modulate the glutamatergic system. One pathway thus links the immune response to alterations across several neurotransmitter systems implicated in psychiatric symptoms [19].

Epidemiological evidence supports this biological plausibility. Systematic reviews and meta‐analyses have repeatedly found a significant association between IgG seropositivity and a range of psychiatric disorders [8, 20, 21]. The association is most consistently replicated for schizophrenia: An early meta‐analysis reported a pooled odds ratio of 2.73 [22], and subsequent methodologically stricter analyses confirmed the link with pooled odds ratios of 1.81–1.91 after adjustment for publication bias [8, 12].

Because toxoplasmosis is common in Iran, the public health implications of such an association would be substantial. A systematic review and meta‐analysis restricted to Iranian studies confirmed a significant association with psychiatric disorders overall (pooled OR = 1.56 for IgG seropositivity) and with schizophrenia specifically (pooled OR = 1.50) [23]; Iranian work has also extended to other psychiatric outcomes, including neurodevelopmental disorders [24]. National pooled estimates, however, can mask regional differences. No such study has been carried out in Golestan province, northern Iran, where a humid climate, intensive agriculture, and local dietary habits may produce transmission dynamics and risk factor profiles unlike those elsewhere in the country. The present study was therefore designed as the first case–control investigation in Gorgan, the provincial capital, to determine the seroprevalence of anti‐*T*. *gondii* IgG and IgM antibodies in hospitalized psychiatric patients and matched healthy controls, and to identify local risk factors for infection.

## 2. Materials and Methods

### 2.1. Study Design, Setting, and Population

A case–control study was conducted in Gorgan, the capital city of Golestan province in northern Iran (Figure 1), between the winter of 2022 and the spring of 2023. The region features a temperate climate with an average annual temperature of 17.6°C and mean annual precipitation of 609 mm. It is located at an elevation of 13.3 m above sea level (coordinates 36°50 ^′^29 ^″^N, 54°25 ^′^58 ^″^E) and experiences warm, humid summers. The case group comprised 100 patients who were consecutively admitted to the inpatient psychiatry department of 5 Azar Hospital, a major teaching and referral hospital affiliated with Golestan University of Medical Sciences.

**Figure 1:**
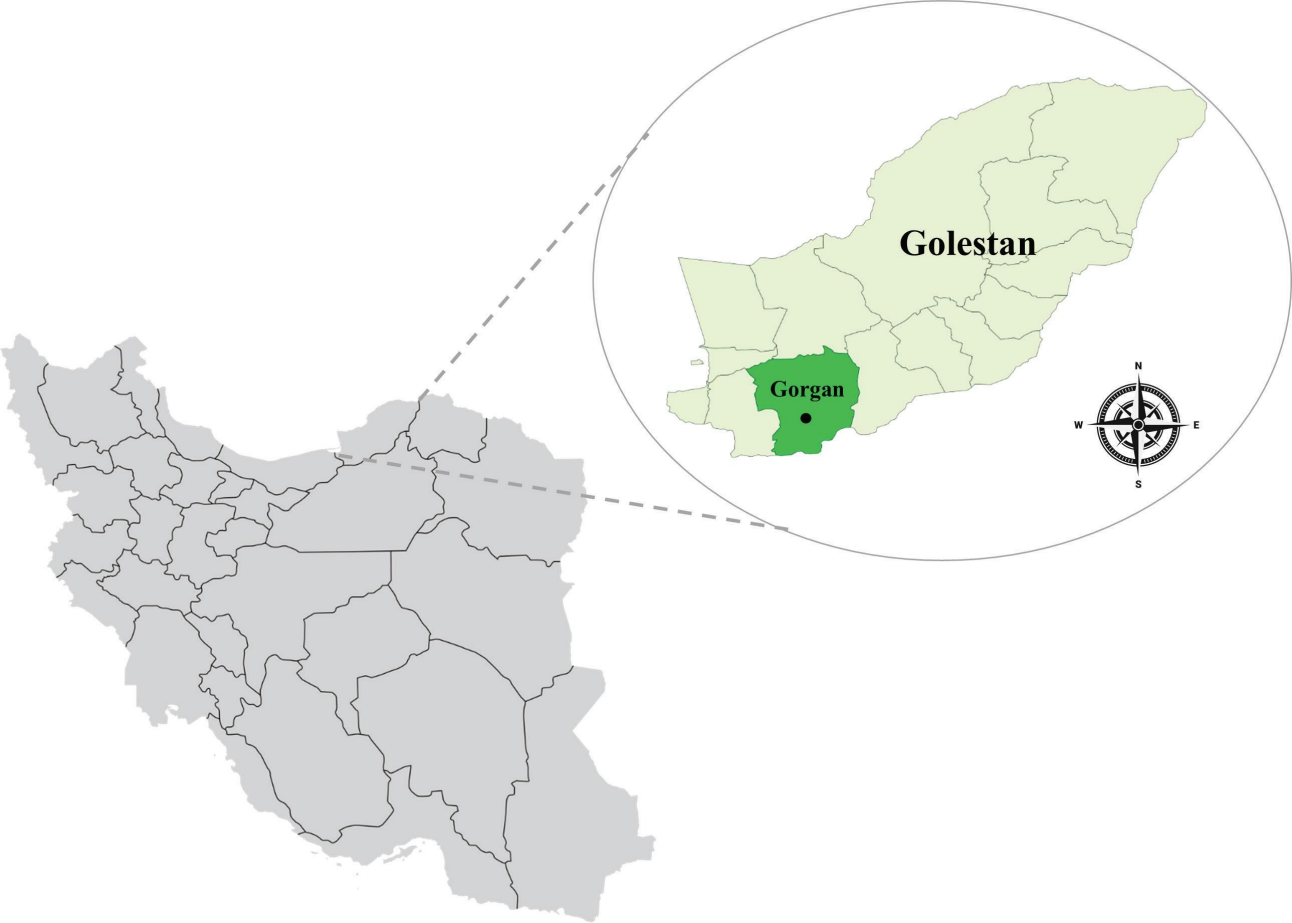
Geographical location of Golestan province and Gorgan City within Iran.

Psychiatric diagnoses were established by the attending board‐certified psychiatrists in accordance with the criteria of the Diagnostic and Statistical Manual of Mental Disorders, fifth edition (DSM‐5) [25]. Diagnoses were entered as free text in the discharge summary rather than as coded categories and were mapped for analysis from the verbatim wording by two members of the research team working independently, with disagreements resolved by a board‐certified psychiatrist. Two rules were fixed before the mapping began: Where more than one diagnosis was recorded, the first‐listed diagnosis determined the category; and records whose wording did not identify a DSM‐5 disorder were not assigned to any category. Six categories were used: schizophrenia spectrum and other psychotic disorders; bipolar and related disorders; depressive disorders; substance‐ or medication‐induced psychiatric disorders; personality disorders; and other psychiatric disorders. This last category comprised attention‐deficit disorder, adjustment disorder, panic disorder with persistent depressive disorder, posttraumatic stress disorder presenting after a suicide attempt, and two records of unspecified mood disorder. The complete verbatim‐to‐category mapping is given as Table S1.

The control group comprised 100 individuals recruited over the same period from consecutive attendees of the Deziani Clinic laboratory. This is a general outpatient laboratory serving the same catchment area as 5 Azar Hospital, and the individuals approached had presented for routine health screening rather than for symptomatic illness. Controls were eligible if they had no current or lifetime psychiatric diagnosis, no psychiatric hospitalization, no psychotropic medication, and no acute or chronic condition under active treatment. Psychiatric history was excluded both by direct interview and by review of the university‐affiliated electronic health record. This control source draws on the same geographical and socioeconomic population that supplies the hospital, which matters when the exposure of interest is environmentally transmitted. To limit selection bias, we used consecutive rather than convenience sampling, restricted recruitment to the catchment area and time window of the cases, administered the same questionnaire and serological protocol to both groups, and kept laboratory personnel blinded to group allocation.

Inclusion criteria for both groups were age of at least 10 years, written informed consent from the participant or a legally authorized representative, and sufficient venous blood for serology. Sixteen participants (nine patients and seven controls) were younger than 18 years; for these, written consent was obtained from a parent or legal guardian and the participant′s own assent was recorded before enrolment. Participants with insufficient sample volume were excluded.

Controls were frequency‐matched to cases 1:1 by sex and 5‐year age group; individual pair matching was not used. Matching was carried out when 98 of the 100 cases had been enrolled and before any serological testing, by recruiting controls into each sex and age group until their number equaled that of the cases; the two cases enrolled subsequently were matched in the same way. The resulting joint distribution of sex and 5‐year age group was identical in the two groups.

### 2.2. Data and Sample Collection

A standardized, structured questionnaire was administered to all participants by trained members of the research team. This instrument collected comprehensive data on demographic characteristics, including age, sex, place of residence (urban/rural), ethnicity, level of education, and occupation. It also gathered information on well‐established risk factors for toxoplasmosis, such as a history of direct contact with cats, frequency of contact with soil (e.g., through gardening or agriculture), and dietary habits, including the consumption of raw vegetables. For each participant, a 5‐mL sample of venous blood was collected using standard phlebotomy procedures. The samples were allowed to clot at room temperature for 30 min, after which serum was separated via centrifugation at 3000 rpm for 15 min. The resulting serum aliquots were transferred to sterile cryotubes and stored at −20°C until they were transported for serological analysis.

### 2.3. Serological Analysis

All serum samples were analyzed for the presence of anti‐*T*. *gondii* IgG and IgM antibodies at the Parasitology and Mycology Laboratory of Golestan University of Medical Sciences. The analysis was performed using commercial enzyme‐linked immunosorbent assay (ELISA) kits (Pishtaz Teb Zaman, Tehran, Iran), following the manufacturer′s detailed protocols. The IgG assay was based on an indirect ELISA principle, where parasite antigens coated on the microplate wells capture specific IgG antibodies from the serum. The IgM assay utilized an antibody‐capture design, where antihuman IgM antibodies on the plate first capture all IgM from the serum, followed by the addition of enzyme‐conjugated *T*. *gondii* antigens to detect specific antiparasite IgM. For both assays, the optical density of the colorimetric reaction was measured at a wavelength of 450 nm using a Stat fax 4200 ELISA reader (Awareness Technology Inc., United States). Samples were classified as seropositive or seronegative based on the cutoff values provided in the kit instructions.

### 2.4. Statistical Analysis

Data were analyzed using IBM SPSS Statistics Version 22.0 (Armonk, New York: IBM Corp) and R Version 4.3. Results are reported as frequencies and percentages, mean ± standard deviation, or median (interquartile range) for skewed variables. Proportions were compared by Pearson chi‐square without continuity correction or by Fisher′s exact test where any expected cell count was below five, and continuous variables by the independent‐samples *t*‐test or Mann–Whitney *U* test. In the diagnosis‐specific comparisons of Table 1, the exact test was used throughout, irrespective of expected cell counts, because every subgroup is small; the row for the whole case group is reported with the chi‐square *p* value so that it remains comparable with Table 2. Wilson intervals were used for proportions.

**Table 1:** Diagnostic composition of the psychiatric case group (*n* = 100) and anti‐*T*. *gondii* seropositivity by DSM‐5 diagnostic category, each compared with the common control group (*n* = 100).

Diagnostic category	*n* (%)	IgG positive, *n* (%)	95% CI (%)	OR vs. controls (95% CI)	90% CI for OR	*p*	IgM positive, *n*
Schizophrenia spectrum and other psychotic disorders	18 (18.0)	7 (38.9)	20.3–61.4	0.73 (0.27–2.00)	0.32–1.70	0.612	0
Schizophrenia	10 (10.0)	6 (60.0)	31.3–83.2	1.63 (0.46–5.76)	0.56–4.70	0.517	0
Schizoaffective disorder	6 (6.0)	1 (16.7)	3.0–56.4	0.31 (0.05–1.95)	0.07–1.45	0.218	0
Delusional disorder or psychotic disorder due to another medical condition	2 (2.0)	0 (0.0)	0.0–65.8	0.23 (0.01–4.81)	0.02–2.94	0.498	0
Bipolar and related disorders	23 (23.0)	8 (34.8)	18.8–55.1	0.62 (0.25–1.55)	0.28–1.34	0.355	0
Manic or mixed index episode	13 (13.0)	4 (30.8)	12.7–57.6	0.53 (0.16–1.75)	0.20–1.45	0.377	0
Depressive disorders	22 (22.0)	8 (36.4)	19.7–57.0	0.66 (0.26–1.68)	0.30–1.44	0.479	0
Major depressive disorder	19 (19.0)	8 (42.1)	23.1–63.7	0.83 (0.32–2.19)	0.37–1.88	0.804	0
Substance/medication‐induced disorders	19 (19.0)	11 (57.9)	36.3–76.9	1.52 (0.58–4.02)	0.68–3.44	0.457	0
Personality disorders	9 (9.0)	2 (22.2)	6.3–54.7	0.38 (0.09–1.66)	0.11–1.30	0.182	1
Other psychiatric disorders	6 (6.0)	2 (33.3)	9.7–70.0	0.63 (0.13–3.08)	0.16–2.39	0.684	0
Diagnosis not classifiable	3 (3.0)	1 (33.3)	6.1–79.2	0.68 (0.09–5.31)	0.12–3.81	1.000	0
All psychiatric patients	100 (100)	39 (39.0)	30.0–48.8	0.72 (0.41–1.27)	0.45–1.16	0.254	1
Healthy controls (reference)	100 (100)	47 (47.0)	37.5–56.7	1 (reference)	—	—	0

**Table 2:** Crude seroprevalence of anti‐*T*. *gondii* antibodies in psychiatric patients and healthy controls.

Antibody	Status	Cases (*n* = 100)	Controls (*n* = 100)	OR (95% CI)	*p*
IgG	Negative	61 (61.0%)	53 (53.0%)	1	0.254
	Positive	39 (39.0%)	47 (47.0%)	0.721 (0.411–1.265)	
IgM	Negative	99 (99.0%)	100 (100.0%)	—	1.000
	Positive	1 (1.0%)	0 (0.0%)	Not estimable	

The primary analysis used unconditional logistic regression, concordant with the frequency‐matched design. Two multivariable models were fitted, distinguished by their dependent variable. Model A takes psychiatric case status as the dependent variable and IgG seropositivity as the exposure, adjusted for smoking, residence, ethnicity, education, and occupation. Model B takes IgG seropositivity as the dependent variable to estimate risk factors for infection, adjusted for age, sex, psychiatric status, residence, education, occupation, and soil contact. Occupation was entered as a set of indicator variables with the “other” occupational group as the reference category. Ethnicity was entered as a single ordinal term in the models reported in Table 3; a version carrying Fars‐referenced indicator variables is reported alongside in the results. In Model B, occupation was represented by the unemployed and homemaker indicators only, the remaining occupational groups forming the reference because the farming category is largely collinear with soil contact; a sensitivity model carrying the full set of occupational indicators is described in the results. Because matching was exact within the joint sex‐by‐age strata, the primary case–control comparison was repeated with adjustment for the matching variables, both by Mantel–Haenszel stratification on the occupied sex‐by‐5‐year‐age cells of the matching grid and by logistic regression carrying age as a continuous term and sex as an indicator.

**Table 3:** Multivariable logistic regression models. Model A estimates the association between IgG seropositivity and psychiatric case status; Model B estimates risk factors for IgG seropositivity.

Variable	B	SE	aOR	95% CI	*p*
Model A—dependent variable: psychiatric case status (*n* = 194)					
IgG seropositivity	−0.278	0.338	0.757	0.390–1.469	0.411
Current smoking	0.962	0.361	2.617	1.289–5.312	0.008
Rural residence	−0.872	0.751	0.418	0.096–1.822	0.246
Ethnicity	−0.229	0.262	0.795	0.476–1.328	0.381
Education (diploma or less)	0.508	0.516	1.663	0.604–4.575	0.325
Contact with soil	2.328	0.684	10.255	2.684–39.185	0.001
Occupation (4 df)	—	—	—	—	0.431
Model B—dependent variable: *T*. *gondii* IgG seropositivity (*n* = 194)					
Psychiatric case status	−0.166	0.355	0.847	0.423–1.698	0.640
Age (per year)	0.074	0.017	1.077	1.042–1.114	< 0.001
Male sex	0.847	0.614	2.332	0.700–7.775	0.168
Rural residence	1.579	0.769	4.850	1.074–21.907	0.040
Education (diploma or less)	0.040	0.462	1.040	0.421–2.572	0.932
Contact with soil	−2.078	0.738	0.125	0.029–0.531	0.005
Unemployed	0.046	0.388	1.048	0.489–2.242	0.905
Homemaker	0.731	0.741	2.078	0.486–8.880	0.324

Secondary analyses compared IgG and IgM seropositivity in each of the six diagnostic categories with the common control group and tested heterogeneity across categories by the Fisher–Freeman–Halton exact test, the expected counts being too small for the chi‐square approximation (Monte Carlo estimate, 200,000 resamples; the chi‐square statistic is reported alongside for reference). Records that could not be assigned to a category are shown as a separate row in Table 1 for completeness but are excluded from that test. These subgroups are small, and the analyses are exploratory; no correction for multiple comparisons was applied. Equivalence was assessed by two one‐sided tests on the log odds ratio against a prespecified margin of 0.641–1.56, derived from the pooled odds ratio for psychiatric disorders in Iranian studies [23]; the upper test was repeated against 1.81 and 2.73, taken from the schizophrenia meta‐analyses [8, 22]. Odds ratios are reported with 95% and 90% confidence intervals, and the narrowest margin at which equivalence was obtained is given for the primary comparison [26, 27]. Within the case group, exploratory comparisons of illness‐course variables (duration since diagnosis, lifetime admissions, electroconvulsive therapy, family history, psychotropic treatment, and lifetime suicide attempt) between seropositive and seronegative patients were also made, restricted to records with complete data. Differential leucocyte counts recorded at admission were used to derive the neutrophil‐to‐lymphocyte ratio, which was compared between groups and by serostatus.

### 2.5. Ethical Considerations

The study was conducted in accordance with the principles stated in the Declaration of Helsinki. The study protocol received full approval from the Research Ethics Committee of Golestan University of Medical Sciences (Ethical Code: IR.GOUMS.REC.1401.035). Written informed consent was obtained from every participant or their legally authorized representative prior to enrollment and data collection. All personal data were anonymized using a coding system to ensure participant confidentiality throughout the study.

## 3. Results

A total of 200 individuals were enrolled, comprising 100 psychiatric inpatients and 100 healthy controls. Both groups comprised 71 men (71.0%) and 29 women (29.0%), and mean age did not differ between them (37.8 ± 12.8 years in cases vs. 37.7 ± 13.1 years in controls, *p* = 0.952).

Demographic and exposure characteristics are shown in Table 4. Compared with controls, patients were more often resident in rural areas (25.0% vs. 13.1%, *p* = 0.033), unemployed (56.0% vs. 37.0%; *p* = 0.008 for the occupational distribution as a whole), and of Turkmen ethnicity (12.0% vs. 2.0%, *p* = 0.017). They were also more often current smokers (49.0% vs. 31.0%, *p* = 0.009) and educated to diploma level or below (90.9% vs. 79.0%, *p* = 0.019). Self‐reported frequent contact with soil was more common among patients (44.8% vs. 14.0%, *p* < 0.001). No participant in either group reported contact with cats, and raw vegetable consumption was universal in both groups; neither variable could be analyzed. Patients were also more often single or divorced (46.0% single and 16.0% divorced vs. 37.0% and 0%, *p* < 0.001) and more often reported washing raw vegetables with detergent rather than with water alone (13.5% vs. 1.0%, *p* < 0.001). Vegetable‐washing method was unrelated to seropositivity (*p* = 1.000); seropositivity was higher in ever married than in single participants (52.5% vs. 28.9%), but this difference did not persist after adjustment for age (aOR = 0.83, 95% CI: 0.37–1.82, *p* = 0.634). Data were missing for a small number of participants, and the corresponding denominators are reduced accordingly: The exposure block (soil contact, cat contact, and raw vegetable intake) was missing for four patients, level of education for one patient, and place of residence for one control.

**Table 4:** Demographic, socioeconomic, and exposure characteristics of psychiatric patients and healthy controls, Gorgan, northern Iran.

Variable	Category	Cases (*n* = 100)	Controls (*n* = 100)	OR (95% CI)	*p*
Sex	Male	71 (71.0%)	71 (71.0%)	Matched	1.000
	Female	29 (29.0%)	29 (29.0%)	Matched	
Age, years	Mean ± SD	37.8 ± 12.8	37.7 ± 13.1	Matched	0.952
Place of residence	Urban	75 (75.0%)	86/99 (86.9%)	1	0.033
	Rural	25 (25.0%)	13/99 (13.1%)	2.205 (1.054–4.614)	
Ethnicity	Fars	79 (79.0%)	85 (85.0%)	1	0.017
	Turkmen	12 (12.0%)	2 (2.0%)	6.456 (1.401–29.753)	
	Sistani	9 (9.0%)	13 (13.0%)	0.745 (0.302–1.838)	
Occupation	Other	41 (41.0%)	52 (52.0%)	1	0.008
	Unemployed	56 (56.0%)	37 (37.0%)	1.920 (1.071–3.439)	
	Employee	3 (3.0%)	11 (11.0%)	0.346 (0.091–1.322)	
Education	Above diploma	9/99 (9.1%)	21 (21.0%)	1	0.019
	Diploma or less	90/99 (90.9%)	79 (79.0%)	2.658 (1.151–6.141)	
Current smoking	Yes	49 (49.0%)	31 (31.0%)	2.139 (1.201–3.809)	0.009
Substance use	Yes	43 (43.0%)	0 (0.0%)	Not estimable	< 0.001
Contact with soil	Yes	43/96 (44.8%)	14 (14.0%)	4.984 (2.491–9.971)	< 0.001
Contact with cats	Yes	0/96 (0.0%)	0 (0.0%)	Not estimable	—
Raw vegetable intake	Yes	96/96 (100%)	100 (100%)	Not estimable	—

The diagnostic composition of the case group, together with seropositivity in each category, is shown in Table 1 and record by record in Table S1. Bipolar and related disorders were the largest category, accounting for 23 patients (23.0%), of whom 22 (22.0%) had bipolar I disorder and 1 (1.0%) cyclothymic disorder. Depressive disorders accounted for 22 (22.0%), of whom 19 (19.0%) had major depressive disorder and 3 (3.0%) presented after a suicide attempt. Schizophrenia spectrum and other psychotic disorders accounted for 18 (18.0%): 10 (10.0%) schizophrenia, 6 (6.0%) schizoaffective disorder, 1 (1.0%) delusional disorder, and 1 (1.0%) psychotic disorder due to another medical condition. The remaining patients had substance‐ or medication‐induced disorders (19, 19.0%), personality disorders (9, 9.0%), or other disorders (6, 6.0%); in three further patients (3.0%), the recorded wording did not identify a DSM‐5 disorder. Within the bipolar group, 11 patients (47.8%) were admitted in a predominantly manic episode and 2 (8.7%) in a mixed episode, giving 13 of 23 (56.5%) with a manic‐predominant index presentation. Polarity was unspecified in the remaining 10 (43.5%), and psychotic features were documented in 10 (43.5%).

There was no significant association between *T*. *gondii* seropositivity and psychiatric status. Anti‐*T*. *gondii* IgG seroprevalence was 39.0% (39/100; 95% CI: 30.0–48.8) in patients and 47.0% (47/100; 95% CI: 37.5–56.7) in controls, giving an odds ratio for psychiatric case status among seropositive individuals of 0.721 (95% CI: 0.411–1.265, *p* = 0.254) (Table 2). Adjustment for the matching variables did not change this: The Mantel–Haenszel odds ratio stratified on the sex‐by‐5‐year‐age matching cells was 0.683 (*p* = 0.278), and the estimate adjusted for age and sex in logistic regression was 0.668 (95% CI: 0.359–1.240, *p* = 0.201). Excluding the single control whose questionnaire recorded past electroconvulsive therapy gave 0.737 (95% CI: 0.419–1.294, *p* = 0.287). One patient (1.0%, a 25‐year‐old with a Cluster B personality disorder who was also IgG positive) and no control tested IgM positive (*p* = 1.000).

Seroprevalence by diagnostic category is presented in Table 1; no category differed significantly from controls. IgG seroprevalence was 38.9% (7/18) in schizophrenia spectrum and other psychotic disorders (OR = 0.73, 95% CI: 0.27–2.00), 34.8% (8/23) in bipolar and related disorders (OR = 0.62, 95% CI: 0.25–1.55), and 36.4% (8/22) in depressive disorders (OR = 0.66, 95% CI: 0.26–1.68). The corresponding figures were 57.9% (11/19) in substance‐ or medication‐induced disorders (OR = 1.52, 95% CI: 0.58–4.02), 22.2% (2/9) in personality disorders, and 33.3% (2/6) in the residual category; one of the three patients whose diagnosis could not be classified was seropositive. Heterogeneity across the six categories was not significant (Fisher–Freeman–Halton *p* = 0.533; chi − square = 4.225, df = 5). Within the spectrum, the 10 patients whose discharge diagnosis was recorded explicitly as schizophrenia gave 60.0% (6/10; OR = 1.63, 95% CI: 0.46–5.76, *p* = 0.517), the six with schizoaffective disorder 16.7% (1/6), and the two with delusional disorder or psychotic disorder due to another medical condition none. With 10 patients, the schizophrenia and spectrum estimates are not statistically distinguishable. The 13 bipolar patients with a manic or mixed index episode gave 30.8% (OR = 0.53, 95% CI: 0.16–1.75), and the 19 patients with major depressive disorder 42.1% (OR = 0.83, 95% CI: 0.32–2.19). The single IgM‐positive participant fell in the personality disorder category; therefore, diagnosis‐specific analysis of IgM was not informative.

Exploratory comparisons within the case group identified no illness‐course variable associated with serostatus. Duration since psychiatric diagnosis, recorded for 92 patients, was a median of 6 [1–15] years in IgG‐positive and 4 [1–10] years in IgG‐negative patients (*p* = 0.275), and the number of lifetime admissions, recorded for 87, was 1 (0–1) versus 0 (0–1) (*p* = 0.118). Current psychotropic treatment (42 patients), lifetime suicide attempt (19), substance use (43), and current smoking (49) were unrelated to serostatus (Fisher′s exact test, all *p* ≥ 0.15; the smallest chi‐square *p* value, for current smoking, was 0.111). Twenty‐three patients (23.0%) had received electroconvulsive therapy at some point, and seropositivity did not differ by this exposure (11/23 vs. 28/77, *p* = 0.340); the number of sessions was documented for only six of them and was therefore not analyzed. Family history of psychiatric illness was recorded as present in seven patients, of whom two were seropositive (*p* = 0.702), a comparison too small to interpret. IgG‐positive patients were older than IgG‐negative patients (median 40 [35–49] vs. 36 [26–45] years, *p* = 0.019), consistent with the age gradient in seroprevalence described below. Differential leucocyte counts were usable for 194 participants. The neutrophil‐to‐lymphocyte ratio was substantially higher in patients than in controls (median 2.00 [1.53–2.56] vs. 1.45 [1.16–1.98], *p* < 0.001), but did not differ by serostatus in either group (patients 1.92 vs. 2.13, *p* = 0.317; controls 1.53 vs. 1.38, *p* = 0.386), and the erythrocyte sedimentation rate likewise did not differ by serostatus (median 11 vs. 11 mm/h, *p* = 0.616). These comparisons were exploratory and unadjusted for multiplicity.

Two multivariable models were fitted and are presented in Table 3. In Model A, with psychiatric case status as the dependent variable, IgG seropositivity was not associated with case status after adjustment (aOR = 0.757, 95% CI: 0.390–1.469, *p* = 0.411). Two other variables in that model were associated with being a psychiatric inpatient rather than with infection: current smoking (aOR = 2.617, 95% CI: 1.289–5.312, *p* = 0.008) and self‐reported soil contact (aOR = 10.255, 95% CI: 2.684–39.185, *p* = 0.001). Entering ethnicity as indicator variables rather than as a single term left these estimates unchanged (IgG seropositivity aOR = 0.783, 95% CI: 0.402–1.525, *p* = 0.472; soil contact aOR = 9.659, 95% CI: 2.490–37.460, *p* = 0.001)

In Model B, with IgG seropositivity as the dependent variable, age was the only consistent independent predictor (aOR = 1.077 per year, 95% CI: 1.042–1.114, *p* < 0.001), and psychiatric status remained nonsignificant (aOR = 0.847, 95% CI: 0.423–1.698, *p* = 0.640). Seroprevalence by age stratum in each study group is shown in Table 5. Seroprevalence rose with age in both groups: monotonically among controls, and among patients with a single flat step between the 31–40 and 41–50 strata (42.9% and 42.1%). The gradient was considerably steeper among controls (aOR = 1.139 per year, 95% CI: 1.079–1.203) than among patients (aOR = 1.043 per year, 95% CI: 1.008–1.079), and this difference was significant (likelihood‐ratio test for the age‐by‐group interaction, chi − square = 8.37, df = 1, *p* = 0.004). The overall inverse point estimate was accordingly confined to older participants. At 40 years of age or younger, the cut point coincides with the boundary of the 5‐year strata used for matching, so that the two groups contain equal numbers seroprevalence was 34.9% (22/63) in patients and 25.4% (16/63) in controls (chi‐square *p* = 0.244, Fisher *p* = 0.332); above 40 years, it was 45.9% (17/37) in patients and 83.8% (31/37) in controls (chi‐square *p* < 0.001, Fisher *p* = 0.001). Given the small strata and the post hoc nature of this comparison, it is not interpreted as evidence of a protective effect. Rural residence was also associated with seropositivity (aOR = 4.850, 95% CI: 1.074–21.907, *p* = 0.040). Soil contact, strongly associated with case status in Model A, was inversely associated with seropositivity in Model B (aOR = 0.125, 95% CI: 0.029–0.531, *p* = 0.005); carrying the full set of occupational indicators in this model left the estimate essentially unchanged (aOR = 0.138, 95% CI: 0.032–0.594, *p* = 0.008), whereas the farming indicator, which overlaps almost completely with reported soil contact, absorbed part of the same signal (aOR = 0.137, 95% CI: 0.020–0.952, *p* = 0.045). This inverse association was confined to the case group (23.3% vs. 47.2%) and absent among controls (50.0% vs. 46.5%, *p* = 1.000).

**Table 5:** Anti‐*T*. *gondii* IgG seroprevalence by age stratum in each study group.

Age stratum (years)	Patients, IgG+/*n*(%)	Controls, IgG+/*n*(%)	Both groups, IgG+/*n*(%)	95% CI (%)
≤ 20	1/10 (10.0)	0/10 (0.0)	1/20 (5.0)	0.9–23.6
21–30	6/18 (33.3)	6/18 (33.3)	12/36 (33.3)	20.2–49.7
31–40	15/35 (42.9)	10/35 (28.6)	25/70 (35.7)	25.5–47.4
41–50	8/19 (42.1)	14/19 (73.7)	22/38 (57.9)	42.2–72.1
> 50	9/18 (50.0)	17/18 (94.4)	26/36 (72.2)	56.0–84.2
Total	39/100 (39.0)	47/100 (47.0)	86/200 (43.0)	36.3–50.0

Equivalence test results for every diagnostic category are given in Table 1. For the case group as a whole, the 90% confidence interval was 0.45–1.16, and the one‐sided tests against 1.56, 1.81, and 2.73 gave *p* = 0.004, *p* = 0.001, and *p* < 0.001; the estimate adjusted for the matching variables gave 0.40–1.12 and *p* = 0.004 against 1.56. Two‐sided equivalence at the 0.641–1.56 margin was not obtained (lower test *p* = 0.341) and was reached only at margins wider than 0.45–2.22. Among the diagnostic categories, the upper 90% confidence limits were 1.34 for bipolar and related disorders, 1.44 for depressive disorders, 1.70 for the schizophrenia spectrum, and 3.44 for substance‐ or medication‐induced disorders, and the one‐sided tests against 1.56 reached significance for bipolar and related disorders (*p* = 0.025), depressive disorders (*p* = 0.035), and personality disorders (*p* = 0.030), but not for the others. For the 10 patients with narrowly defined schizophrenia, the upper 90% limit was 4.70.

## 4. Discussion

In the present study, we found no significant difference in *T*. *gondii* IgG seroprevalence between psychiatric inpatients (39.0%) and matched healthy controls (47.0%) (OR = 0.721, 95% CI: 0.411–1.265, *p* = 0.254). This is the first case–control study of this association in Gorgan, northern Iran. Seropositivity increased with age in both groups, from 5.0% at 20 years or younger to 72.2% above 50 years. Adjustment for the matching variables left the estimate unchanged (OR = 0.668, 95% CI: 0.359–1.240)

We found no excess of seropositivity in any diagnostic category. In the schizophrenia spectrum as a whole, seroprevalence was 38.9% (OR = 0.73); in the 10 patients whose diagnosis was recorded explicitly as schizophrenia, it was 60.0% (OR = 1.63, 95% CI: 0.46–5.76). Torrey et al. [22] reported a pooled OR of 2.73 for schizophrenia, and later meta‐analyses reported ORs of 1.81–1.91 [8, 12]. Our schizophrenia estimate is nominally compatible with those figures and equally compatible with the null, because with 10 patients, the interval spans an approximately eightfold range; we therefore draw no inference from it in either direction. The spectrum estimate, which rests on 18 patients, lies below unity and is closer to that of Cano et al. [28] in Colombia, who found a lower seroprevalence in patients with schizophrenia (20.2%) than in their environmental controls (31.3%). In bipolar disorder, we found 34.8% (OR = 0.62) and in the manic‐predominant subgroup 30.8% (OR = 0.53), whereas Sutterland et al. [8] reported an elevated pooled OR for bipolar disorder. Depressive disorders gave 36.4% (OR = 0.66). The only category with a higher rate than the controls was substance or medication‐induced disorder (57.9%, OR = 1.52), and this difference was not significant; Alvarado‐Esquivel et al. [29] also found no difference between patients with substance‐use disorders and controls (10.1% vs. 9.4%). Seroprevalence did not differ across the six categories (*p* = 0.533). With 6–23 patients per category, the intervals are wide. The upper 90% confidence limit was 1.34 for bipolar and related disorders and 1.44 for depressive disorders, both below the pooled Iranian estimate of 1.56; for the schizophrenia spectrum, it was 1.70, which excludes the pooled meta‐analytic odds ratio of 1.81 but not 1.56. Associations of the magnitude reported for these disorders are thus inconsistent with our data, whereas smaller associations, and the estimate derived from the 10 patients with narrowly defined schizophrenia (upper 90% limit 4.70), are not excluded.

Within the patient group, seropositivity was not related to the duration of illness, the number of admissions, current medication, lifetime suicide attempt, substance use, or smoking. Kürkçü et al. [30] reported the same in 155 Turkish patients with schizophrenia: Brief Psychiatric Rating Scale scores and illness duration were higher in seropositive patients, but the differences were not significant. That study did, however, find seroprevalence itself to be significantly higher in patients than in controls, so it supports the association at the group level while agreeing with us that serostatus does not track clinical course. Alvarado‐Esquivel et al. [29] also found no association with the clinical or behavioral characteristics of their patients. Andreou et al. [31] studied 765 Norwegian patients with severe mental illness and did find a difference, but in the opposite direction, with lower psychotic symptom scores in seropositive patients who had been ill for 10 years or more.

Our control seroprevalence (47.0%) was much higher than in the studies that reported the strongest positive associations. Yolken et al. [32] reported 36.8% in patients against 11.1% in controls in the United States, Wang et al. [33] 16.5% against 6.0% in China, and Cetinkaya et al. [34] 66% against 22% in Turkey. When half of the general population is already seropositive by middle age, the difference in exposure between patients and controls becomes small and a moderate association is difficult to detect. Two other differences may also matter. Those studies included only patients with schizophrenia [32–35], whereas in our series, only 18% had a schizophrenia spectrum diagnosis and 10% a diagnosis recorded explicitly as schizophrenia. Our controls were laboratory attendees rather than blood donors or community volunteers, which would tend to lower rather than raise their seroprevalence, so this cannot explain the negative result. The lower rate in patients was confined to those over 40 years of age, in whom control seroprevalence reached 83.8%; with 37 participants per group in this stratum, we regard this as a chance finding rather than evidence of protection. We also found no evidence that a single transmission route explained the pattern, since no participant in either group reported contact with cats and soil contact was related to patient status but not to seropositivity.

Results within Iran also vary. Montazeri et al. [23] reported pooled ORs of 1.56 for psychiatric disorders and 1.50 for schizophrenia, but the individual studies differ widely. Abdollahian et al. [4] found a positive association in Mashhad, whereas Saraei‐Sahnesaraei et al. [36] found none in Qazvin, where seroprevalence was 55.3% in patients with schizophrenia and 50.9% in controls. The control rate in Qazvin is close to ours, and the result there was also negative. Studies in humid provinces with high background prevalence therefore tend to be negative, and those in drier provinces positive. Golestan belongs to the first group.

These regional differences affect burden estimates. Nessim et al. [37] applied meta‐analytic odds ratios to seroprevalence data by continent and estimated that 20.4% of schizophrenia and 27.3% of bipolar disorder could be attributed to *T*. *gondii*. Such estimates assume that the association is the same everywhere. Our data do not support that assumption for a high‐prevalence region.

Cano et al. [28] compared 100 patients with schizophrenia in Colombia with both biological relatives and neighborhood‐matched controls and found seropositivity lower in patients (20.2%) than in relatives (45.7%) or neighborhood controls (31.3%). They also performed GRA6‐based serotyping and found the low‐virulence Type III serotype to predominate among seropositive participants, although its distribution did not differ between cases and controls within their sample. Strain type nevertheless affects the strength of the host immune response, and the mechanisms proposed for psychiatric effects, namely, dopamine dysregulation, neuroinflammation, and kynurenine pathway activation, depend on it. Iranian isolates have been reported as mainly Types I and II, but no genotyping data are available for Golestan province and we did not genotype our samples. This should be examined in future studies in this region.

Our serological results were recorded only as positive or negative, so antibody levels could not be compared between the two groups. IgM was positive in only one of 200 participants. Almost all infections were therefore latent rather than recent. A second line of evidence points the same way. Patients in this series carried a clear systemic inflammatory signature relative to controls, the neutrophil‐to‐lymphocyte ratio being about 38% higher, yet that signature was unrelated to *Toxoplasma* serostatus within either group, and the erythrocyte sedimentation rate did not vary by serostatus either. The sample was thus able to detect an inflammation‐related difference where one existed; it simply did not locate that difference along the axis of *Toxoplasma* exposure. A negative epidemiological result does not exclude an effect of the parasite on the brain; dopamine dysregulation and chronic neuroinflammation are well documented [13]. It suggests instead that such an effect is difficult to detect by case–control comparison in a population where most people have been exposed.

Several methodological limitations should be considered when interpreting these findings. First, the diagnostic subgroups are small (*n* = 6–23), and their confidence intervals are correspondingly wide. Diagnoses were mapped from free‐text discharge summaries rather than structured clinical interviews or dimensional symptom severity scales. Second, serological assessment relied on a single qualitative indirect ELISA without quantitative IgG titers, antibody avidity indices, or direct measures of central nervous system cyst burden; nevertheless, universal IgM negativity and stable systemic inflammatory markers (NLR and ESR) consistently indicate latent rather than active infection. Third, controls were recruited from an outpatient clinic rather than the general community, which may introduce selection bias, and cases were restricted to hospitalized inpatients at a single tertiary center, limiting generalizability to settings with different background seroprevalences or parasite lineages. Finally, the cross‐sectional detection of IgG antibodies marks lifelong cumulative exposure, precluding inferences regarding the temporal sequence or causal nature of the relationship.

## 5. Conclusion

In this high‐prevalence region of northern Iran, *T*. *gondii* IgG seropositivity was not associated with psychiatric disorder, either in the pooled patient group or in any DSM‐5 diagnostic category, including the schizophrenia spectrum and manic‐predominant bipolar disorder. Point estimates lay below unity for five of the six categories and none reached significance; the exceptions were substance‐ or medication‐induced disorder and, within the schizophrenia spectrum, the 10 patients with narrowly defined schizophrenia, whose estimate lay above unity with an interval spanning an approximately eightfold range. The confidence intervals exclude an association as large as the pooled Iranian odds ratio of 1.56, but not a smaller or inverse one. Seroprevalence was determined principally by age, consistent with lifelong cumulative environmental exposure in an endemic setting.

## Author Contributions

Hamidreza Zakerzadeh Khoshroudi: conceptualization, project administration, software, writing—review and editing. Najmeh Shahini: conceptualization, methodology, visualization, writing—review and editing. Rasool Mohammadi: data curation, methodology, software, writing—review and editing. Farideh Tohidi: methodology, visualization, writing—review and editing. Aieneh Hagieh Pangh: data curation, methodology, writing—review and editing. Seyed Mohammad Mousavi: data curation, software, writing—original draft, writing—review and editing. Mitra Sharbatkhori: conceptualization, data curation, funding acquisition, methodology, project administration, software, supervision, visualization, writing—original draft, writing—review and editing.

## Funding

This study was supported by Golestan University of Medical Sciences (10.13039/501100008091; 112778).

## Disclosure

The authors reviewed and edited the output and take full responsibility for the content of the publication.

## Conflicts of Interest

The authors declare no conflicts of interest.

## Supporting Information

Additional supporting information can be found online in the Supporting Information section.

## Supporting information


**Supporting Information 1** Table S1: Complete record‐by‐record mapping of the verbatim free‐text discharge diagnoses of the 100 psychiatric inpatients to the six DSM‐5–based diagnostic categories used in the analysis.

**Supporting Information 2** Dataset S1: De‐identified participant‐level dataset for all 200 participants (100 patients and 100 controls), containing every variable used in the analyses, together with a variable dictionary.

## Data Availability

The data that support the findings of this study are available from the corresponding authors upon reasonable request.
