# Supplementary material for: Seroepidemiology of Toxoplasma gondii Infection in Psychiatric Inpatients and Healthy Controls in North Iran: A Case–Control Study
Source: J Parasitol Res. 2026 Oct 14;2026:2530719. doi: 10.1155/japr/2530719 (PMC13662829; doi:10.1155/japr/2530719)
Supplement: Supplementary file 1 [file JAPR-2026-2530719-s001.docx]

**Supplementary Table S1.** Verbatim discharge diagnosis, number of patients, number seropositive for anti-*Toxoplasma gondii* IgG, and the DSM-5 diagnosis assigned for analysis (psychiatric case group, n = 100).

| **Diagnosis as recorded in the discharge summary** | **n** | **IgG +** | **DSM-5 diagnosis assigned** |
| --- | --- | --- | --- |
| **Schizophrenia spectrum and other psychotic disorders** | **18** | **7** |  |
| schizophrenia | 7 | 5 | Schizophrenia |
| chronic schizophrenia | 1 | 1 | Schizophrenia |
| chronic schizophernia | 1 | 0 | Schizophrenia |
| residual schizophernia | 1 | 0 | Schizophrenia |
| schizoaffective | 6 | 1 | Schizoaffective disorder |
| dellusion disorder | 1 | 0 | Delusional disorder |
| psychotic disorder due to AMC | 1 | 0 | Psychotic disorder due to another medical condition |
| **Bipolar and related disorders** | **23** | **8** |  |
| Bipolar | 2 | 1 | Bipolar I disorder, polarity unspecified |
| BJD [sic; read BID] | 3 | 1 | Bipolar I disorder, polarity unspecified |
| BID + PF | 3 | 1 | Bipolar I disorder with psychotic features, polarity unspecified |
| BMD | 1 | 0 | Bipolar I disorder, polarity unspecified |
| Bipolar (manic phase) | 3 | 0 | Bipolar I disorder, manic |
| Bipolar (manic episode) | 1 | 0 | Bipolar I disorder, manic |
| bipolar with manic | 1 | 0 | Bipolar I disorder, manic |
| BID manic episode currently in acute phase | 1 | 1 | Bipolar I disorder, manic |
| BID manic episode + PF | 1 | 0 | Bipolar I disorder, manic, with psychotic features |
| BID mianc episode + PF [sic] | 1 | 0 | Bipolar I disorder, manic, with psychotic features |
| BID manic episode with PF | 1 | 1 | Bipolar I disorder, manic, with psychotic features |
| BID multiple episode currently in acute manic phase with psychotic feature | 1 | 0 | Bipolar I disorder, manic, with psychotic features |
| manic episode with PF | 1 | 0 | Bipolar I disorder, manic, with psychotic features |
| BID manic episode with micxed psychosis [sic] | 1 | 1 | Bipolar I disorder, mixed, with psychotic features |
| BID with mix phase + PF | 1 | 1 | Bipolar I disorder, mixed, with psychotic features |
| cyclothymia | 1 | 1 | Cyclothymic disorder |
| **Depressive disorders** | **22** | **8** |  |
| MDD | 17 | 7 | Major depressive disorder |
| MDD with psychotic feature | 1 | 1 | Major depressive disorder with psychotic features |
| MDD + illness anxiety disorder | 1 | 0 | Major depressive disorder (first-listed rule) |
| suicide attempt | 2 | 0 | Depressive presentation after suicide attempt |
| suicide | 1 | 0 | Depressive presentation after suicide attempt |
| **Substance/medication-induced disorders** | **19** | **11** |  |
| substance induced psychotic disorder | 7 | 6 | Substance-induced psychotic disorder |
| substance induced psychosis | 5 | 1 | Substance-induced psychotic disorder |
| substance induced | 6 | 3 | Substance-induced disorder, not further specified |
| substance induced mood disorder | 1 | 1 | Substance-induced mood disorder |
| **Personality disorders** | **9** | **2** |  |
| border line | 4 | 0 | Borderline personality disorder |
| border line + suicide attempt | 1 | 0 | Borderline personality disorder (first-listed rule) |
| Antisocial Personality Disorder | 1 | 0 | Antisocial personality disorder |
| personality disorder cluster B | 1 | 1 | Cluster B personality disorder |
| personality disorder | 1 | 1 | Personality disorder, not further specified |
| personality disorder + substance induced | 1 | 0 | Personality disorder, not further specified (first-listed rule) |
| **Other psychiatric disorders** | **6** | **2** |  |
| ADD | 1 | 0 | Attention-deficit disorder |
| Adjust disorder | 1 | 1 | Adjustment disorder |
| Panic Disorder + Disthymia | 1 | 0 | Panic disorder with persistent depressive disorder (first-listed rule) |
| PTSD + suicide attempt | 1 | 0 | Post-traumatic stress disorder presenting after suicide attempt (first-listed rule) |
| mood disorder | 1 | 1 | Unspecified mood disorder |
| mood disorder due to AMC | 1 | 0 | Unspecified mood disorder due to another medical condition |
| **Diagnosis not classifiable** | **3** | **1** |  |
| ?! [no diagnosis entered] | 2 | 1 | Not assignable — no diagnosis recorded |
| behavioral disturbance due to I.D | 1 | 0 | Not assignable — behavioural disturbance in intellectual disability |
| **All psychiatric patients** | **100** | **39** |  |

**Note.** Diagnoses were entered as free text in the discharge summary rather than as coded categories. They were mapped from the verbatim wording by two members of the research team working independently, with disagreements resolved by a board-certified psychiatrist. Two rules were fixed before the mapping began and are applied without exception: where more than one diagnosis is recorded, the first-listed diagnosis determines the category (marked “first-listed rule” below); and records whose wording does not identify a DSM-5 disorder are not assigned to any category and are reported separately. Three records (3.0% of the case group) could not be assigned to a DSM-5 category: two in which no diagnosis had been entered and one recorded as behavioral disturbance in the context of intellectual disability. One of the three is IgG seropositive. These records are counted in the denominator of the whole case group throughout the manuscript but are not included in any of the six diagnostic categories, and they are excluded from the test of heterogeneity across categories.
